# *Lobesia botrana*: A Biological Control Approach with a Biopesticide Based on Entomopathogenic Fungi in the Winter Season in Chile

**DOI:** 10.3390/insects13010008

**Published:** 2021-12-21

**Authors:** Fabiola Altimira, Nathalia De La Barra, Paulo Godoy, Juan Roa, Sebastián Godoy, Nancy Vitta, Eduardo Tapia

**Affiliations:** 1 Laboratory of Entomology and Biotechnology, Instituto de Investigaciones Agropecuarias, INIA La Platina, Santiago 8831314, Chile; fabiola.altimira@inia.cl (F.A.); godoygonzalez.s@gmail.com (S.G.); nvitta@inia.cl (N.V.); 2 Programa Nacional de *Lobesia botrana* Región Metropolitana, Servicio Agrícola y Ganadero, Santiago 9170009, Chile; nathalia.delabarra.m@gmail.com; 3 Extension Department, Instituto de Investigaciones Agropecuarias, INIA La Platina, Santiago 8831314, Chile; paulo.godoy@inia.cl (P.G.); jroa@inia.cl (J.R.)

**Keywords:** *Lobesia botrana*, entomopatogenic fungi, formulation, biocontrol, *Beauveria pseudobassiana*, *Metarhizium roberstsii*

## Abstract

**Simple Summary:**

*Lobesia botrana*, also known as the European grapevine moth, is one of the main pests that affect grapes. In Chile, this type of moth is classified as a quarantine pest, which requires fumigating the fruit with methyl bromide to prevent the immature stages of the pest from being strained and reaching the export-destination countries. In the fields, the larvae of this moth feed on grapes, which can introduce diseases such as *Botrytis cinerea*, thereby increasing the costs of managing the crop. One way to control this pest is to use entomopathogenic fungi on the winter pupae to reduce moth populations in the spring. In the present study, six fungi were characterized, formulated, and evaluated. The selected strains RGM 2184 and RGM 678 were evaluated in two regions of Chile during two seasons. These strains reached maximum efficiencies of 80% and 88%, respectively. Therefore, the use of entomopathogenic fungi is an environmentally friendly alternative to control *L. botrana* and reduce the use of chemical pesticides.

**Abstract:**

*Lobesia botrana* (Denis and Shiffermüller) (Lepidoptera: Tortricidae) is one of the main pests that affect the production and export of table grapes in Chile. Because this pest has quarantine status, the fruit must be fumigated with methyl bromide, which reduces the fruit’s export competitiveness in the destination market. In the present study, to help resolve this issue, six native entomopathogenic fungi were identified through multilocus analysis, including three *Beauveria pseudobassiana* and three *Metarhizium robertsii*. These fungi were evaluated in the laboratory to control *L. botrana* in its pupal stage in a silk cocoon and compared against a biological control product. Formulations with additional carbon sources improved the performance of the fungi. The treatments with outstanding performance contained the fungal strains *B. pseudobassiana* RGM 2184 and *M. robertsii* RGM 678. These strains were evaluated in the field during the winter season in two different regions of the country; the strains reached maximum efficacies of 80% and 88%, respectively, at 21 days post first application. Therefore, entomopathogenic fungi can contribute to reducing pupal populations in winter, thereby decreasing the moth population in spring–summer.

## 1. Introduction

*Lobesia botrana* (Denis and Shiffermüller) (Lepidoptera: Tortricidae), commonly known as the European grapevine moth, is one of the main pests affecting Chilean table grape exports. This pest has been reported in Europe, Africa, Asia, and South America (Argentine and Chile) and was eradicated in California, USA, in 2016 [1,2]. In Chile, this moth has been a quarantine pest under official control by the Agricultural and Livestock Service (SAG, acronym in Spanish) since April 2008 [3]. At present, this moth is distributed in the northern to southern areas of Chile between Atacama and Araucania. Under climate change-induced increases in temperature, this moth has the potential to expand further south and may threaten crops established in this area, such as blueberries, other native berries, and new grape crops, which could promote the settlement of moth populations. Variations in voltinism can be generated under the same environmental conditions, including a fourth flight of the pest over a long summer in Chile, as reported in Europe [4,5]. The main damage from this pest is caused by its larvae, which penetrate and feed on the grape berries, thus promoting the development of diseases such as *Botrytis cinerea* [6] and *Aspergillus* section Nigri [7]. Such damage increases the costs of managing orchards since a control strategy for moths, as well as fungicidal products for diseases associated with larval damage, must be considered. In Chile, another adverse consequence of this moth is fumigation of the fruit with methyl bromide, which is required for export to markets with restrictions on this pest. The infected fruit is impacted by the fumigation process, which requires increased temperatures. Under this process, the quality and postharvest time of the fruit are reduced, and the environmental humidity is increased, which promotes the incidence of diseases.

Currently, it is necessary to control pests in an environmentally friendly way. The incorporation of sustainable technologies, such as the sterile insect technique [8], natural enemies [9], and biopesticides [10,11,12], as well as monitoring the applications of such technologies, remains key for integrated pest management (IPM). Biopesticides based on entomopathogenic fungi (EPF) can be used during the winter diapause of the pest. The advantages of these biopesticides are based on their different mechanisms of action for killing arthropods [13,14,15]. When the EPF spore adheres to the surface of the insect, an appressorium develops and eventually penetrates the cuticle of the insect, thus allowing the entry and development of the hypha, which secretes different metabolites, enzymes, and peptides. This secretome enables the establishment of the EPF and the formation of blastospores that colonize the insect and begin a new cycle of the EPF [15]. One example of this process is the EPF strain *B. pseudobassiana* RGM 1747, which was able to penetrate the *L. botrana* silk cocoon and ultimately reach the pupa [16]. This is an important factor because the silk cocoon is refractory to chemical pesticides, and commercial products are not available for controlling pupae in the winter. Therefore, to verify whether the use of a biopesticide based in EPFs as a control tool for *L. botrana* in the pupal stage in winter, in this work, the goal was to evaluate the biopesticide efficacy of EPF in the field for the control of *L. botrana* pupae on two different grape cultivars, ‘Red Globe’ and ‘Cabernet Sauvignon’, in two different regions of the central area of Chile in winter. We used three EPF strains of *Beauveria* sp. and three of *Metarhizium* sp., which were isolated from southern Chile, enabling them to survive and function under low temperatures and high humidity conditions. The selected strains were *B. pseudobassiana* RGM 2184 and *M. robertsii* RGM 678, which reached efficacies of 80% and 88%, respectively, at 21 days post the first application during the winter season in the field within the central area of the country. The results show that this control strategy can help to decrease populations of this moth during their first flight.

## 2. Materials and Methods

### 2.1. Biological Material

The pupae of *L. botrana* were obtained from the National Program of *Lobesia botrana* (PNLb: Programa Nacional de *Lobesia botrana* in Spanish, Santiago, Chile) of the Agriculture Service and Livestock (Servicio Agrícola y Ganadero in Spanish, Santiago, Chile). The EPF strains *B. pseudobassiana* RGM 1747; *Beauveria* sp. RGM 2184 and RGM 2186; and *Metharizium* sp. RGM 672, RGM 674, and RGM 678 were obtained from the Bank of the Chilean Collection of Microbial Genetic Resources of Agricultural Research Institute (Instituto de Investigaciones Agropecuarias in Spanish, Santiago, Chile). All selected strains were from southern Chile and native to areas with low temperatures and high humidity.

### 2.2. DNA Extraction, PCR Conditions, and Sequencing

The conidial DNA of the different strains of EPF was extracted using a Quick DNA Fungal/Bacterial Kit (Zymo Research, CA, USA) following the manufacturer’s instructions. The partial sequences amplified by PCR for *Beauveria* were the B-locus intergenic-region genomic sequence (*bloc*), translation elongation factor 1-α (*tef*), DNA-dependent RNA polymerase II largest subunit (*rpb1*), and DNA-dependent RNA polymerase II second largest subunit (*rpb2*) loci [17], while those for *Metarhizium* were the *tef, rpb1, rpb2*, and β-tubulin (*btuB*) loci [18,19]. All amplicons were sequenced at Macrogen (Seoul, South Korea). The DNA sequences determined for these genetic markers were submitted to the GENBANK Nucleotide Sequence Database under the accession numbers shown in Table S1.

### 2.3. Multilocus Sequence Analysis (MLSA) of the EPF Strains

For the strains of *Beauveria*, concatenated alignments were performed with MUSCLE for the *bloc, tef, rpb1*, and *rpb2* loci in 34 strains [20]. For the strains of *Metarhizium*, such alignments were performed for the *tef, rpb1, rpb2*, and *btuB* loci in 28 strains. In all phylogenetic analyses, evolutionary histories were inferred using the neighbor-joining method [21]. The evolutionary distances were computed using the Tamura 3-parameter method [22]. The strengths of the internal branches of the resulting trees were statistically evaluated by bootstrap analysis [23]. Finally, evolutionary analyses were conducted in MEGA7 [24].

### 2.4. In Vitro Insecticide Activity of Buffer Suspension (B) of the Wettable Powder (WP) and Inverse Emulsion (IE) Formulations of EPF against Pupae with Silk Cocoons of L. botrana

The EPF strains were cultivated in Petri dishes with a potato dextrose agar (PDA) medium (BD, USA) for 72 h at 25 °C. The surfaces of the PDA cultures were gently rinsed to detach the spores under sterile conditions. The suspension of spores in PBS (phosphate-buffered saline) was collected and filtered using three layers of cheesecloth. The EPF strains were formulated as inverse emulsions (IEs) with and without carbon-source potato starch to evaluate the effects of nutrient addition on the efficacy of the formulation. Inverse emulsion with the carbon source was carried out using a mixture of the aqueous phase and oil phase in a 1:1 ratio. The aqueous phase contained a 95% spore suspension solution (10^8^ spores/mL), 4.25% glycerol (Sigma-Aldrich, St. Louis, MO, USA), and 0.75% Silwet L-77 Ag (Momentive Performance Material INC, Nueva York, NY, USA). In the formulation with an additional carbon source, we added 15% potato starch to 100% of the aqueous phase. In both formulations, with and without a carbon source, the oil phase contained 96% vegetable oil and 4% Tween 20 (Sigma-Aldrich, St. Louis, MO, USA) [24]. The spore suspensions in PBS and the spore IE solution were diluted 10 times in water to obtain 10^7^ spores/mL (=10^7^ UFC/mL). The viability of the final suspension was validated by the CFU count (data not shown). The efficacy evaluation was carried out using a static Potter spray tower (Burkard Manufacturing Co., Ltd., Rickmansworth, UK). In total, 1 mL of each resuspension was sprayed over 10 pupae with silk cocoons in a Petri dish containing a wet paper towel to maintain humidity. As a biopesticide control, a product composed of *Beauveria bassiana* (5 × 10^4^ CFU/mL), *Metarhizium anisopliae* (5 × 10^4^ CFU/mL), and *Paecilomyces lilacinus* (1 × 10^8^ CFU/mL) was used. Only water was used as the absolute control. The experiment used 5 replicates for every treatment. The Petri dishes were incubated at 25 °C and 70–90% relative humidity and the growth of the EPF was monitored every 24 h for 14 d. The strains with high efficacy and the ability to grow in a liquid culture medium (data not shown) were selected and formulated as a wettable powder (WP) supplemented with a carbon source following the methodology described by Tapia et al. [25]. The efficacy evaluation of the WP was carried out under the same conditions as described above.

### 2.5. Field Efficacy Assays

The assays were developed using two different cultivars in two regions of Chile: the table grape *V. vinifera* ‘Red Globe’ vineyard at the INIA La Platina research station in the Metropolitan Region and *V. vinifera* ‘Cabernet Sauvignon’ vineyards in the O’Higgins Region. The trials were conducted for two winter seasons under natural environmental conditions from July to September for two consecutive years, 2018 and 2019. In the metropolitan region, which has an average temperature and relative humidity of 14 ± 3 °C and 64% ± 11%, respectively, the field trial during the winter season was arranged in a randomized block design with six treatments, each with four vines featuring four replicates (6 × 4 × 4). To infest the vines, 100 pupae with silk cocoons per plant were deposited under the rhytidome in a tulle bag according to the methodology described by Altimira and colleagues [16]. In the O’Higgins Region, which has an average temperature and relative humidity of 15 ± 3 °C and 64 ± 11%, respectively, the field trial in the winter season was arranged under the same design (6 × 4 × 4) but with natural infestation previously determined by the PNLb of SAG. Six treatments were evaluated in these regions: (1) *B. pseudobassiana* RGM 2184 IE, (2) *B. pseudobassiana* RGM 2184 WP, (3) *M. robertsii* RGM 678 IE, (4) *M. robertsii* RGM 678 WP, (5) biopesticide control, and (6) absolute control (water). However, during the winter season in the metropolitan region in 2018, the treatments were carried out as follows: (1) *B. pseudobassiana* RGM 2184 WP, (2) *M. robertsii* RGM 678 WP, (3) biopesticide control, and (4) absolute control. All treatments featured three applications that occurred on the first, seventh, and fourteenth days. The EPF concentration of the treatments was 10^7^ CFU/mL. The applications were performed with a motorized backpack sprayer, and wetting was performed with a volume of 500 mL per vine. Each treatment was inspected 7 days post-application (dpa). During the inspection, ten pupae were removed from under the rhytidome and deposited on a plate containing a humid paper disk. Plates were incubated at 25 °C for three weeks to identify dead pupae (mycosis or lack of mobility when they were stung) and determine the treatment efficacy.

### 2.6. Statistical Analyses

The in vitro efficacy was determined for homogeneous populations using the arrangement developed by Abbott [26]. The efficacy in the field assay was determined for nonhomogeneous populations using the arrangement of Henderson and Tilton [27]. The percentages of efficacy during the experiments determined by Abbott and Henderson and Tilton were analyzed by ANOVA LSD Fisher test (α = 0.05). In parallel, to determine the best treatments in field trials, the interactions of microorganisms with the formulations and the efficacy of the treatments in exterminating the pest were analyzed over time through generalized linear mixed models (GLMM) and compared using an LSD Fisher test. For these analyses, the dependent variable was efficacy, the random variables were microorganisms, formulation, and days. The fixed effect was the treatments, and the selected distribution was Poisson. All experiments were analyzed and graphed using InfoStat^®^ (2020 version) and GraphPad Prism 9.0.0 (121)^®^ (2020 version), respectively.

## 3. Results

### 3.1. Molecular Identification of the EPF Strains

**Figure 1:**
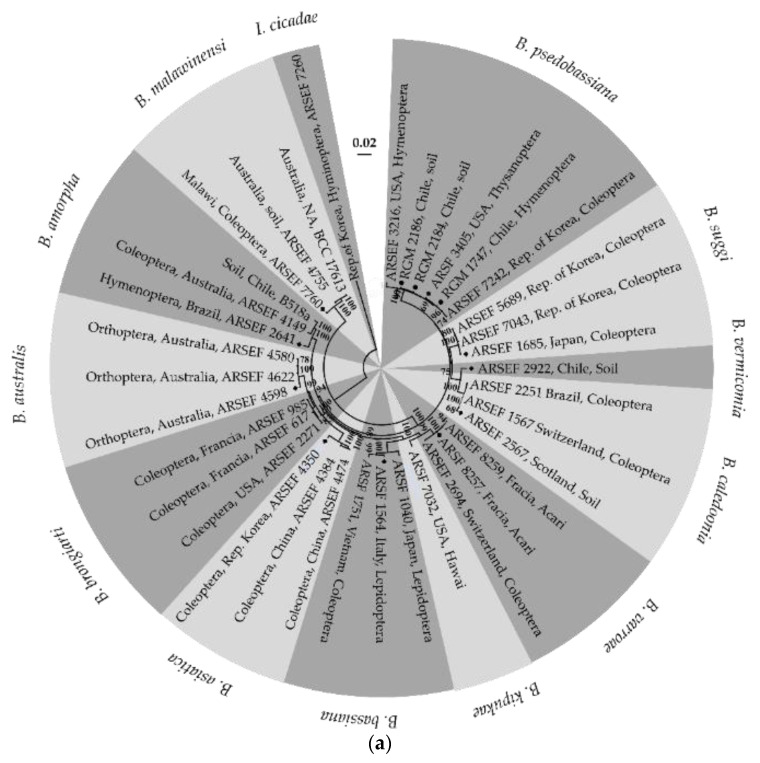

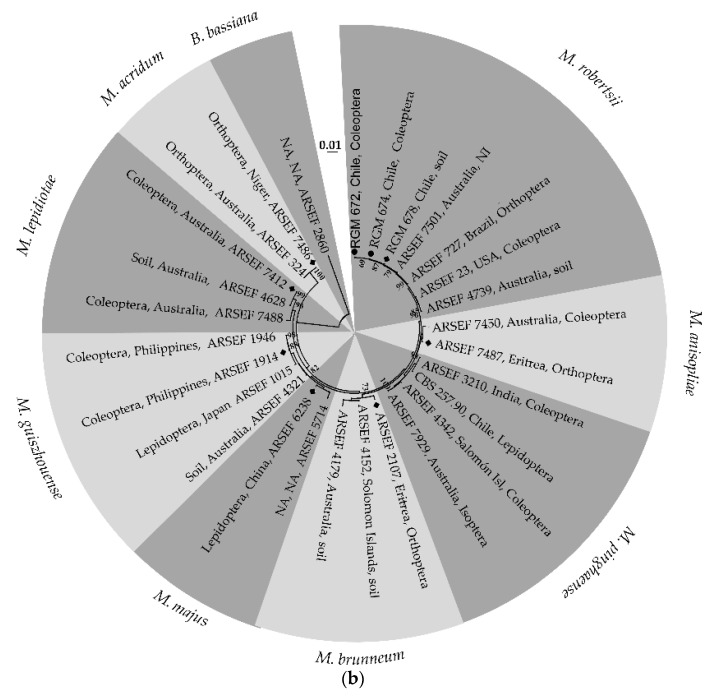
Phylogenetic tree of the *Beauveria* and *Metarhizium* spp. strains based on multilocus analysis. The tree is drawn to scale, with branch lengths measured by the number of substitutions per site. (**a**) Phylogenetic tree of *Beauveria* spp. strains obtained with the neighbor-joining method for *bloc*, *tef*, *rpb1*, and *rpb2*. (**b**) Phylogenetic tree of *Metharizium* spp. strains obtained with the neighbor-joining method for *tef*, *rpb1*, *rpb2*, and *btuB*. Bootstrap values of 50% are labeled above the appropriate internodes. The type strains are indicated with diamonds, and our strains are indicated with circles. NA, not available.

In the first stage, EPF strains isolated from Chilean areas with low temperatures and high humidity levels were identified through the MLSA. The *Beauveria* strains RGM 2184, RGM 2186, and RGM 1747 were grouped in the same clade as the strain type *B. pseudobassiana* ARSEF 7242 and other *B. pseudobassiana* strains (Figure 1a). The *Metarhizium* strains RGM 672, RGM 674, and RGM 678 share the same clade as the type strains *M. robertsii* ARSEF 727 and other *M. robertsii* strains (Figure 1b). Therefore, strains RGM 2184, RGM 2186, and RGM 1747 were identified as *B. pseudobassiana*, and strains RGM 672, RGM 674, and RGM 678 were identified as *M. robertsii*.

### 3.2. In Vitro Efficacy Assay Selection of Formulated EPF

**Figure 2:**
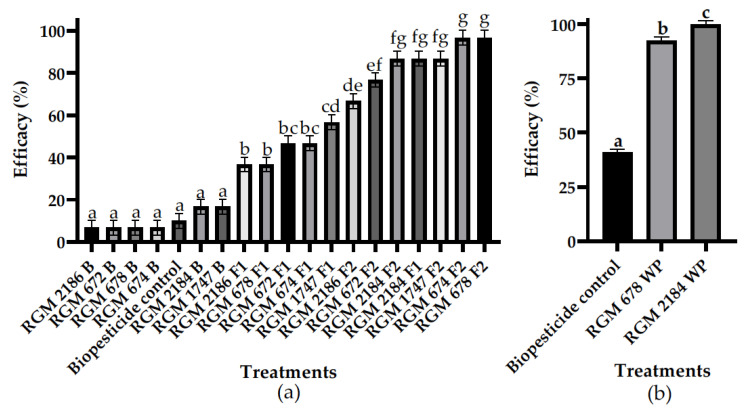
EPF strain in vitro efficacy assay at 7 d. (**a**) Efficacy evaluation of EPF-strain spore suspensions in buffer (B) and formulated as inverse emulsions with (F2) and without (F1) an additional source of energy; (**b**) efficacy evaluation of EPF-strain spores formulated as wettable powder (WP). The letters on the bars represent the comparison using an LSD test (α = 0.05) at 7 dpa for the EPF. Different letters in each Figure represent statistically significant differences.

The efficacy of the EPF strains resuspended in the PBS solution (B) and formulated IE with (F2) or without (F1) an additional carbon source was evaluated against pupae in silk cocoons. All EPF-formulated supplemented carbon resources showed efficacies over 50% at 7 dpa (Figure 2a). *M. robertsii* RGM 674 F2 and RGM 678 F2 stood out and reached efficacies of 97% at 7 dpa. Furthermore, *B. pseudobassiana* RGM 1747 F2 and RGM 2184 F1 reached 87% efficacy at 7 dpa, and the latter strain achieved acceptable performance without a supplementary carbon source. At 14 dpa, almost all the EPFs reached 100% efficacy, except those that were not formulated (Figure S1). Additionally, *B. pseudobassiana* RGM 2184 and *M. robertsii* RGM 678 were formulated as WP and evaluated against pupae with silk cocoons in an in vitro assay (Figure 2b). The biopesticide control reached an efficacy of 41% at 7 dpa. RGM 2184 WP reached an efficacy of 100%, and RGM 678 WP reached 93% efficacy at 7 dpa.

### 3.3. Field Efficacy Assay of the Formulated EPF

**Figure 3:**
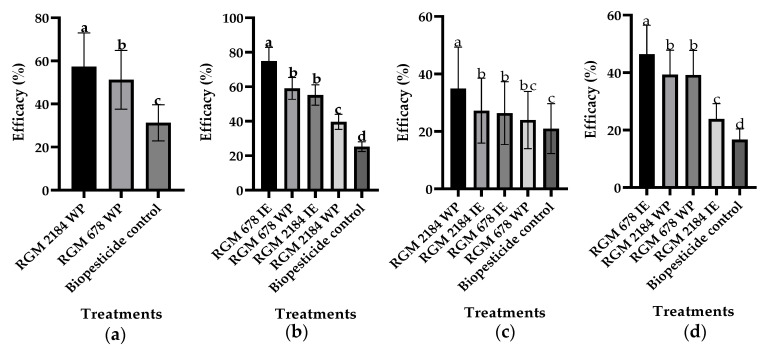
Comparison in the field of different treatments using GLMM. The graph presents the fitted values. The formulated strains RGM 678 and RGM 2184 were tested in an inverse emulsion (IE) and a wettable powder (WP). All formulations were supplemented with a source of energy. (**a**,**b**) Field trials that were carried out in the metropolitan region (Platina) and correspond to 2018 and 2019, respectively. (**c**,**d**) Field trials that were carried out in the O’Higgins region (Placilla) and correspond to 2018 and 2019, respectively. The Y-axis represents the adjusted efficacy (%) obtained from the GLMM. The x axis provides the treatments. The bars represent the means of the treatments, and the whiskers represent the estimated errors. The letters on the bars represent a comparison using an LSD test (α = 0.05) of the different treatments evaluated. Different letters in each Figure represent statistically significant differences.

**Figure 4:**
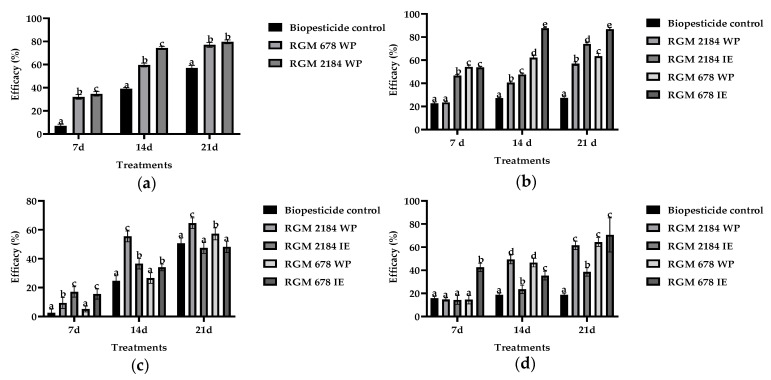
Comparison of the treatment efficacies in the field (ANOVA). The efficacy of strains RGM 678 and RGM 2184 formulated as inverse emulsion (IE) and wettable powder (WP) were evaluated in the field. All the formulation were supplemented with a source of energy. (**a**,**b**) Field trials that were carried out in the metropolitan region (Platina) during 2018 and 2019, respectively. (**c**,**d**) Field trials that were carried out in the O’Higgins region (Placilla) during 2018 and 2019, respectively. The y axis represents the efficacy (%). The X-axis presents the period of evaluation at 7, 14, and 21 days post first application. The bars represent the means of the treatments, and the whisker represent the estimated errors. The letters on the bars represent a comparison using an LSD test (α = 0.05) of the different treatments evaluated. Different letters in each Figure represent statistically significant differences.

The efficacies of strains RGM 2184 and RGM 678 formulated against artificially infested pupae with silk cocoons from the table grape *V. vinifera* ‘Red Globe’ (Metropolitan Region) during the winter seasons were evaluated through GLMM analysis. In 2018, the RGM 2184 WP treatment reached 57% efficacy, followed by RGM 678 WP with 51% efficacy and the biopesticide control with 31% efficacy (Figure 3a). In 2019, RGM 678 IE treatment reached 75% efficacy, followed by RGM 678 WP with 59%, RGM 2184 IE with 55%, RGM 2184 WP with 40%, and the biopesticide control with 25% (Figure 3b). Additionally, the treatment efficacy was compared through an ANOVA (followed by LSD Fisher test) at 7, 14, and 21 days post first application (dpfa) (Figure 4). The WP formulations of RGM 2184 and RGM 678 in the winter season of 2018 exceeded 50% efficacy at 14 dpfa, ultimately reaching 80% and 77% efficacy, respectively, at 21 dpfa. The biopesticide control reached 57% efficiency at 21 dpfa (Figure 4a). In 2019, the WP and IE formulations of RGM 678 reached 54% efficacy at 7 dpfa and 62% and 88% efficacy at 14 dpfa, respectively, and these results were maintained until 21 dpfa. The WP and IE formulations of RGM 2184 reached 57% and 74% at 21 dpfa, respectively, while the biopesticide control reached 27% (Figure 4b).

The efficacies of the formulated EPFs against naturally infested pupae with silk cocoons from the wine grape *V. vinifera* ‘Cabernet Sauvignon’ (O’Higgins region) during consecutive winter seasons were evaluated through GLMM analysis. In 2018, the RGM 2184 WP treatment reached 35% efficacy, followed by RGM 2184 IE with 27%, RGM 678 IE with 26%, RGM 678 WP with 24%, and the biopesticide control with 21% (Figure 3c). In 2019, the RGM 678 IE treatment reached 46% efficacy followed by RGM 2184 WP and RGM 678 WP (both with 39%), RGM 2184 IE with 24%, and the biopesticide control with 17% (Figure 3d). The treatment efficacy was compared using ANOVA (LSD Fisher test) at 7, 14, and 21 dpfa. The RGM 2184 WP passed 50% efficacy at 14 dpfa and reached 65% efficacy at 21 dpfa. Complementarily, the RGM 678 WP formulation reached 57% at 21 dpfa, while the biopesticide control reached 51% at 21 dpfa (Figure 4c). In the 2019 season, RGM 2184 WP reached 49% efficacy at 14 dpfa and 62% at 21 dpfa. The WP and IE formulations of RGM 678 reached 64% and 71% efficacy, respectively, at 21 dpfa. The biopesticide control reached 19% efficacy at 14 and 21 dpfa (Figure 4d).

## 4. Discussion

Entomopathogenic fungi are specialized microorganisms able to infect and reduce natural arthropod populations. This capability could be used as an alternative to chemical insecticides for pest control. EPF strains from the genera *Metarhizium*, *Beauveria*, *Cordyceps*, and *Akanthomyces* are the most commonly used for pest control because these strains are relatively easy to massify and have a wide range of hosts [15]. In this study, *Beauveria* and *Metarhizium* strains adapted to low temperatures were evaluated to find the best candidate for *L. botrana* pupae control in the winter season. These strains were identified as *B. pseudobassiana* and *M. robertsii*. Strains of *B. pseudobassiana* have been isolated from Coleoptera (South Korea), Hymenoptera (Chile, USA), Thysanoptera (USA), and soil samples (Chile) (Figure 1a). Strains of *M. robertsii* have been isolated from Orthoptera (Brazil), Coleoptera (USA and Chile), soil samples (Australia and Chile), and Lepidoptera (Argentina) (Figure 1b). The different areas and sources of isolation suggest that these species are cosmopolitan and that their arthropod targets may not be specific to the family, genus, or species level.

The in vitro assays carried out in this study showed that the formulations of the EPF strains helped to significantly increase efficacy against the pupae of *L. botrana*. Additionally, in most cases, supplementing the formulation with a carbon source enhanced the efficacy of the EPF strains (Figure 2). These results indicate that optimization of the formulation is key for the biocontrol activity of the EPF. Another study carried out in vitro efficacy tests of *M. robertsii* strains, and the unformulated strains showed an efficacy of 80–99.8% against *L. botrana* pupae without silk cocoons at 7 days after incubation [28]. These strains obtained efficacies superior to those of our strains, even without the formulation. The apparently lower efficacy of our strains could be because our tests were carried out in pupae with silk cocoons during the winter diapause stage. In this period of the year, the silk cocoon cover layer is robust [29]; therefore, the fungus must have the ability to adhere to highly hydrophobic tissue along with secreting an enzymatic battery to penetrate the tissue. Our results suggest that the formulation of EPFs with surfactants and nutrients promotes the adherence and secretion of hydrolytic enzymes, respectively, which can help overcome this barrier.

In the field trials, the best treatments were the RGM 2184 WP and RGM 678 IE treatments during the 2018 (Figure 3a,c) and 2019 (Figure 3b,d) seasons, respectively. Based on our experience, we recommend the use of IE formulations in rainy winter seasons due to their greater adherence to the rhytidome. Moreover, the oily consistency of such formulations prevents the product from being washed away by the action of rain during the season. However, WP formulations are recommended in the winter–spring transition period because the inverse emulsion formulation can affect the cotton bud tissue of the vine. Additionally, the efficacy of the formulated EPFs increased according to the number of applications that were made. On average, an increase of 50% in efficacy was obtained between the first and third applications (Figure 4). These results suggest that an *L. botrana* management program in climatological zones similar to those of the metropolitan region and O’Higgins region of Chile (Mediterranean-type climate) with a similar level of infestation requires three consecutive applications every 7 d.

Despite the interest in biological pest control strategies, few scientific works have evaluated the efficacy of EPF on *L. botrana* under field conditions. On this issue, a study by Cozzi et al. [7] determined the mortality of six EPF isolates on *L. botrana* larvae. The best strain, *B. bassiana* ITM 1559, showed a mortality of 55%. Furthermore, in field trials, the incidence of clusters injured by *L. botrana* larvae was significantly reduced under treatment with this strain compared to the untreated control. Likewise, 51% efficacy of the strain *B. pseudobassiana* RGM 1747 was obtained against *L. botrana* pupae in urban areas during winter [16]. This work laid the foundations for the current study in which the WP formulation *B. pseudobassiana* RGM 2184 and IE formulation of the *M. robertsii* RGM 678 achieved maximum efficacy levels of 80% and 88%, respectively, in field trials performed in two regions during two seasons.

These results demonstrate that the EPF biopesticide can contribute to reducing pupal populations in winter and subsequently decrease the moth population in spring–summer. Additionally, EPFs act in a period that is not affected by the preventive or control-based applications of fungal diseases.

In future studies, it is recommended to test EPF mixtures that can withstand spring–summer temperatures to extend the activity of EPF to different stages of development of the pest. Finally, it is recommended to include compatibility tests with fungicides commonly used for grapevine diseases in the selection of new EPFs. 

## Data Availability

All amplified DNA fragments from the entomopathogenic fungal isolates were registered in NCBI GenBank and are presented in Table S1. The isolates were deposited in the Chilean Collection of Microbial Genetic Resources, International Depository Authority, under access numbers RGM 672, RGM 674, RGM 678, RGM 1747, RGM 2184, and RGM 2186.

## Supplementary Materials

The following are available online at https://www.mdpi.com/article/10.3390/insects13010008/s1, Table S1: EPF strain, host, country of collection, and GenBank accession numbers; Figure S1: In vitro efficacy assay at 14 d.
