# Supplementary material for: Lobesia botrana: A Biological Control Approach with a Biopesticide Based on Entomopathogenic Fungi in the Winter Season in Chile
Source: Insects. 2021 Dec 21;13(1):8. doi: 10.3390/insects13010008 (PMC8780027; doi:10.3390/insects13010008)
Supplement: Supplementary file 1 [file insects-13-00008-s001.zip › insects-1478927-supplementary/Table S1.pdf]

insects

MDPI

Table S1. EPF strain, host, country of collection, and GenBank accession number

Strain	Identification	Host (Order: Family)	Country	*bloc*	*btuB*	*tef*	*rpb1*	*rpb2*
RGM 1747	*B. pseudobassiana*	*Naupactus xanthographus* (Hymenoptera: Curculionidae)	Chile	MH048643	ND	MH048642	MH048640	MH048641
RGM 2184	*B. pseudobassiana*	Soil	Chile	MZ350774	ND	MZ350762	MZ350764	MZ350772
RGM 2186	*B. pseudobassiana*	Soil	Chile	MZ350775	ND	MZ350763	MZ350765	MZ350773
RGM 672	*M. robertsii*	*Polistes gallicus* (Coleoptera: Vespidae)	Chile	ND*	MZ350766	MZ350756	MZ350769	MZ350759
RGM 674	*M. robertsii*	*Phyllopertha horticola* (Coleoptera: Scarabaeidae)	Chile	ND	MZ350767	MZ350757	MZ350770	MZ350760
RGM 678	*M. robertsii*	Soil	Chile	ND	MZ350768	MZ350758	MZ350771	MZ350761

*ND: not determined.
