# Supplementary figures and images for: Lobesia botrana: A Biological Control Approach with a Biopesticide Based on Entomopathogenic Fungi in the Winter Season in Chile
Source: Insects. 2021 Dec 21;13(1):8. doi: 10.3390/insects13010008 (PMC8780027; doi:10.3390/insects13010008)

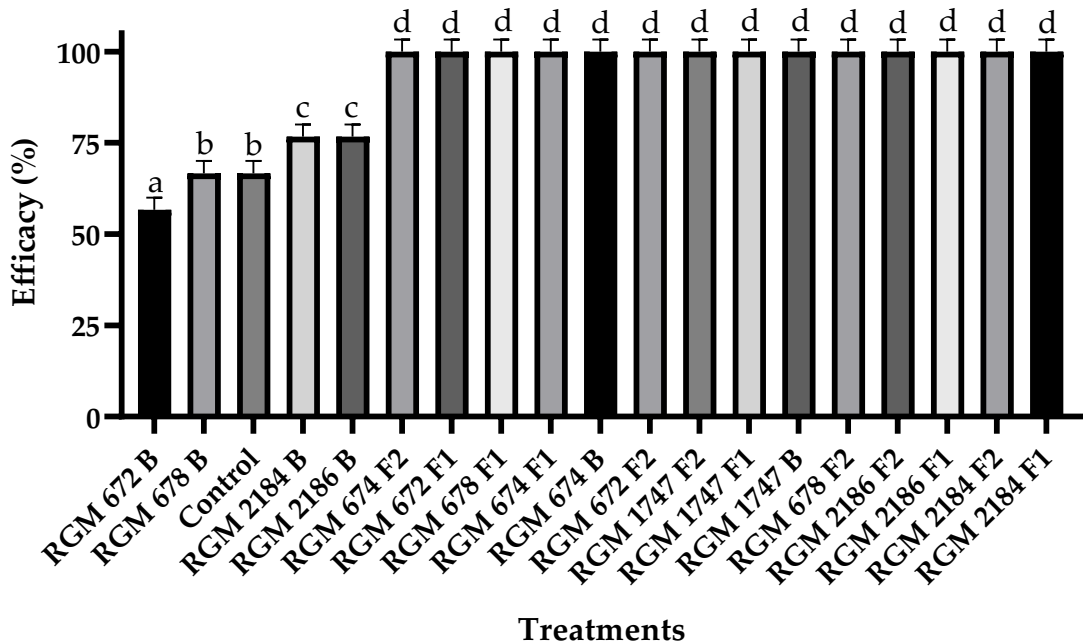

Supplement: Supplementary file 1 [file insects-13-00008-s001.zip › insects-1478927-supplementary/Figure S1.pdf]
